# Dexmedetomidine alleviates lipopolysaccharide-induced lung injury in Wistar rats

**DOI:** 10.18632/oncotarget.17899

**Published:** 2017-05-16

**Authors:** Xuetao Yan, Xiaoli Cheng, Liwen Zhou, Xianghu He, Wenzhong Zheng, Hu Chen

**Affiliations:** ^1^ Department of Anesthesiology, Shenzhen Bao’an Maternity and Child Health Hospital, Shenzhen, 518100, China; ^2^ Department of Pharmacy, Shenzhen Bao’an Maternity and Child Health Hospital, Shenzhen, 518100, China; ^3^ Department of Anesthesiology, Xiangyang Central Hospital, Xiangyang, 441021, China; ^4^ Department of Anesthesiology, Zhongnan Hospital of Wuhan University, Wuhan, 430071, China

**Keywords:** dexmedetomidine, lung injury, inflammation, oxidative stress, Nrf2

## Abstract

This study aimed to investigate the protective effects of dexmedetomidine on lipopolysaccharide (LPS)-induced lung injury in Wistar rats. 24 female Wistar rats were randomly assigned into 3 groups (*n* = 8): a control group, a LPS-challenged group, and a LPS plus dexmedetomidine group. Inflammation, oxidative stress, Nrf2/Keap1, and Akt signal were determined. The results showed that LPS caused inflammation and oxidative stress via increasing pro-inflammatory cytokines and oxidative products. Dexmedetomidine treatment alleviated inflammation and oxidative stress in LPS-challenged rats. Nrf2/Keap1 was inhibited and Akt signal was activated in the lung after exposure to LPS, while dexmedetomidine activated Nrf2/Keap1, which further mediated expressions of antioxidant genes. In conclusion, dexmedetomidine alleviated inflammatory response and oxidative stress in LPS-induced lung injury in rats via influencing Nrf2/Keap1 signal.

## INTRODUCTION

Acute respiratory failure syndromes are devastating disorders characterized by non-cardiogenic pulmonary edema with high morbidity and mortality [1]. Acute lung injury is a major causes of acute respiratory failure and many animal models have demonstrated that inflammation and oxidative stress involve in the pathophysiological mechanisms [2, 3]. In a cecal ligation and puncture reproduced rat acute lung injury model, lung exhibits marked inflammation and oxidative injury evidenced by the increased infiltration of leukocytes, generation of pro-inflammatory cytokines, and oxidative products [4]. Thus, anti-inflammatory and antioxidant agents have been widely used to alleviate acute lung injury in animal models. For example, pyrrolidine dithiocarbamate has been indicated to be a potential therapeutic strategy for acute lung injury via improving inflammatory and oxidative status [5].

Dexmedetomidine, a central α-2 agonist like Clonidine, has been widely used in the field of medicine for lung disorders arranging from lung injury to lung cancers [6, 7]. Meanwhile, dexmedetomidine suppresses systemic inflammation and oxidative stress [8], suggesting a protective role in inflammation and oxidative stress associated diseases. Thus, the aim of this study was to investigate the effect and potential mechanism of dexmedetomidine on lipopolysaccharide-induced lung injury in Wistar rats.

## RESULTS

### Inflammation

Expressions of IL-1β, IL-4, IL-6, IL-10, IL-17, and TNF-α in the lung after exposure to LPS were determined via RT-PCR. The results showed that LPS caused inflammatory response in the lung evidenced by the over-expressions of IL-1β, IL-6, IL-17, and TNF-α (*P <* 0.05; Table 1). Dexmedetomidine markedly downregulated IL-1β and TNF-α expressions (*P <* 0.05), indicating an anti-inflammatory effect of dexmedetomidine.

**Table 1:** Dexmedetomidine alleviated LPS-induced inflammatory response in the lung of rats

Item	Cont	LPS	Dex
IL-1β	1.00 ± 0.08c	1.52 ± 0.11a	1.31 ± 0.15b
IL-4	1.00 ± 0.12	1.06 ± 0.13	1.11 ± 0.14
IL-6	1.00 ± 0.11b	1.37 ± 0.15a	1.36 ± 0.18a
IL-10	1.00 ± 0.13	1.15 ± 0.16	1.03 ± 0.11
IL-17	1.00 ± 0.11b	1.41 ± 0.12a	1.24 ± 0.16ab
TNF-α	1.00 ± 0.09b	1.66 ± 0.17a	1.21 ± 0.12b

Data are presented as mean ± SEM. The values having different superscript letters were significantly different (*P* < 0.05).

### Oxidative stress

**Figure 1:**
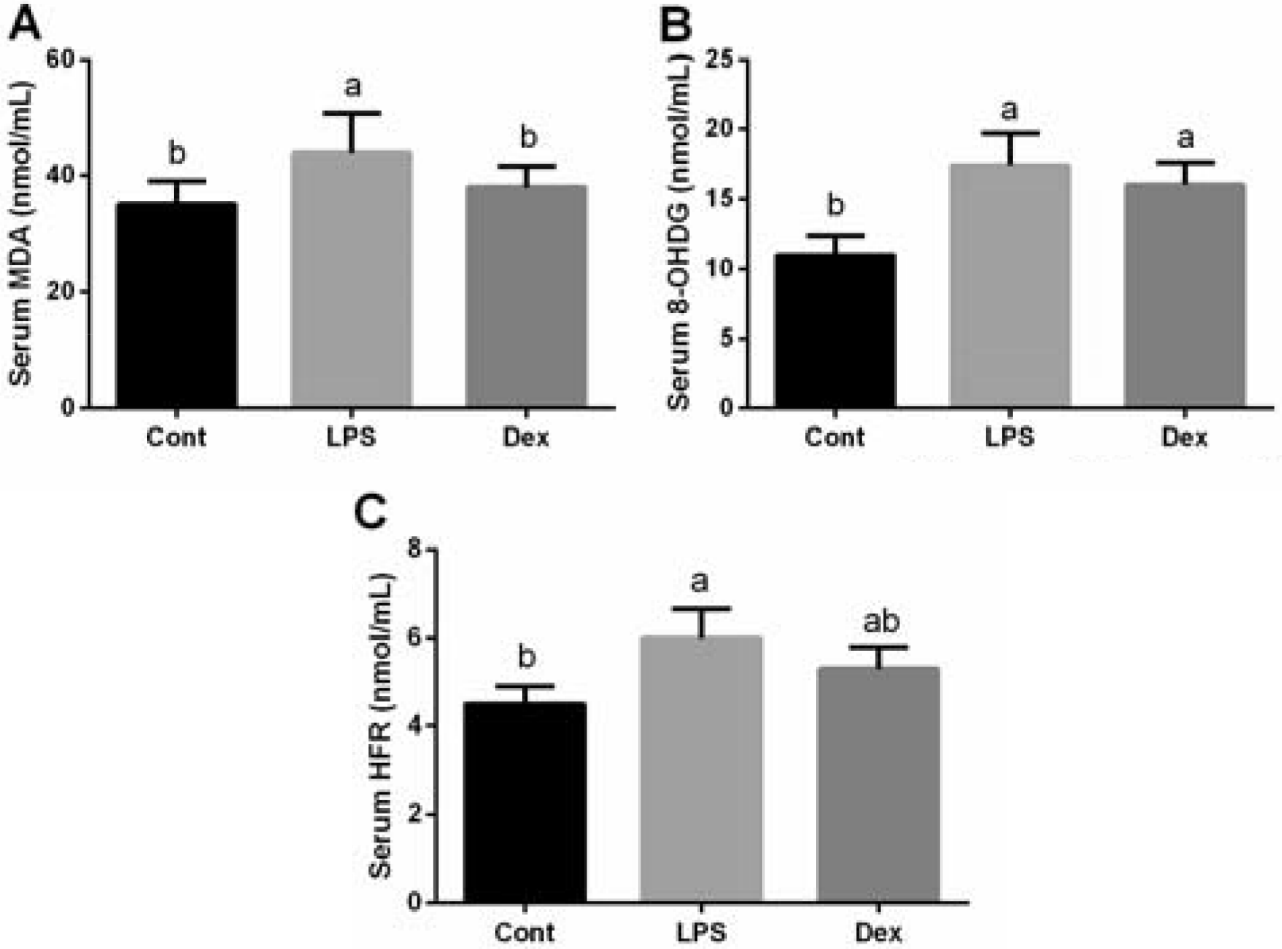
Dexmedetomidine alleviated LPS-induced oxidative stress in the serum of rats Data are presented as mean ± SEM. The values having different superscript letters were significantly different (*P* < 0.05).

Malondialdehyde (MDA), 8-hydroxydeoxyguanosine (8-OHDG), and hydroxyl free radical (HFR) are three major oxidative products from lipid, DNA, and protein, respectively. Serum MDA, 8-OHDG, and HFR were significantly higher in the LPS group than that in the control group (*P <* 0.05; Figure 1). Although dexmedetomidine treatment failed to affect serum 8-OHDG and HFR abundances, MDA production was significantly reduced in Dex group (*P <* 0.05; Figure 1).

**Figure 2:**
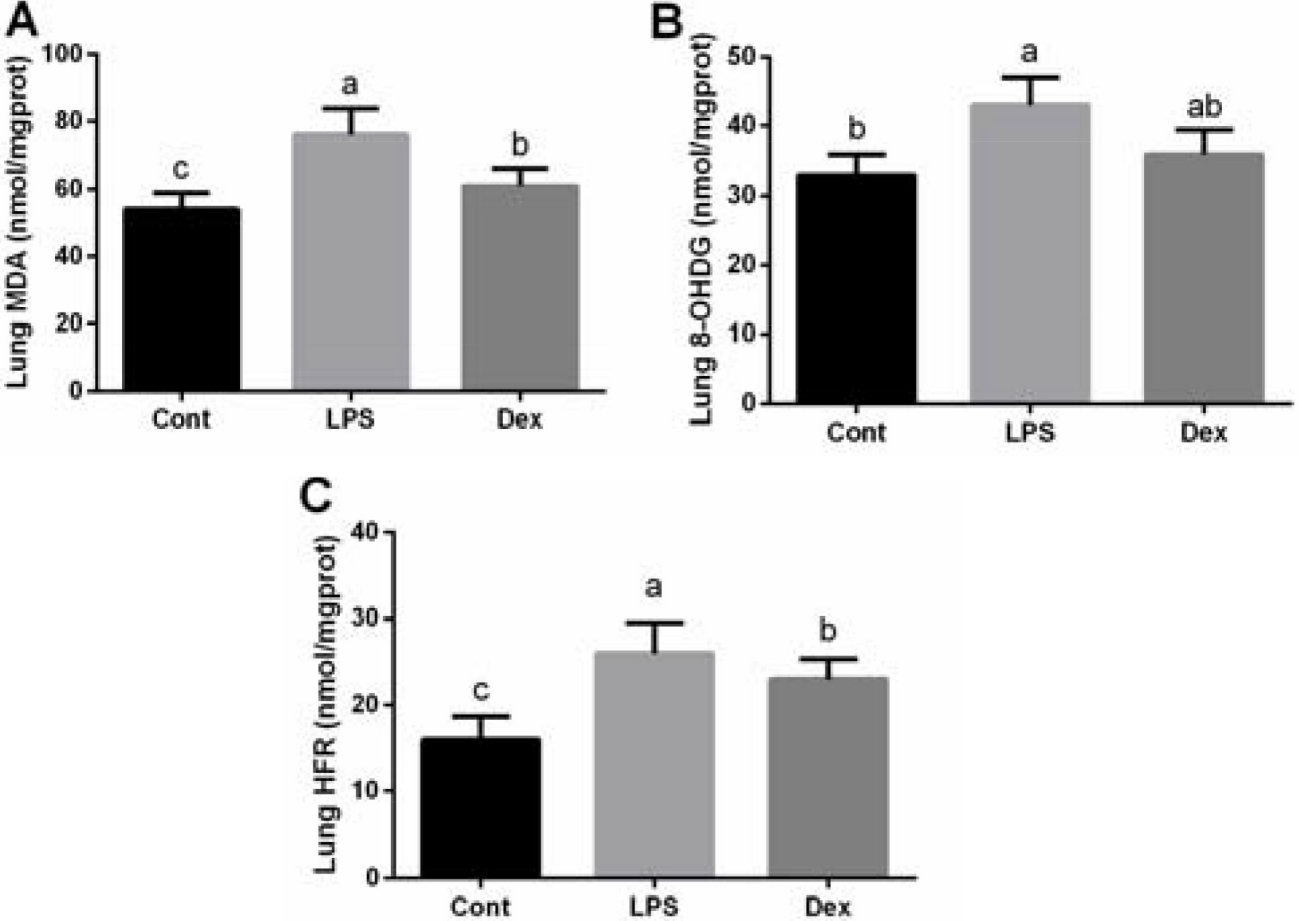
Dexmedetomidine alleviated LPS-induced oxidative stress in the lung of rats Data are presented as mean ± SEM. The values having different superscript letters were significantly different (*P* < 0.05).

Abundances of MDA, 8-OHDG, and HFR in the lung were shown at Figure 2. Similarly, lung also exhibited oxidative stress evidenced by the enhanced MDA, 8-OHDG, and HFR concentrations (*P <* 0.05; Figure 2). Dexmedetomidine treatment markedly reduced MDA and HFR generation, suggesting that dexmedetomidine alleviated LPS-induced lipid and protein oxidation.

### Expressions of antioxidant genes

Expressions of antioxidant genes (i.e. NQO1, NQO2, CAT, GPX1, GPX2, SOD1, and SOD2) in the lung were further investigated to explore the mechanism of oxidative stress caused by LPS in rats. mRNA abundances of NQO1, GPX1, GPX2, and SOD1 were markedly lower in LPS-challenged rats (*P <* 0.05; Table 2). Dexmedetomidine treatment significantly upregulated SOD1 expression (*P <* 0.05; Table 2). Meanwhile, dexmedetomidine tended to increase NQO1, GPX1, and GPX2 expressions, but the differences were insignificant (*P* > 0.05).

**Table 2:** Dexmedetomidine alleviated LPS-induced downregulation of antioxidant genes in the lung of rats

Item	Cont	LPS	Dex
NQO1	1.00 ± 0.09a	0.79 ± 0.08b	0.88 ± 0.11ab
NQO2	1.00 ± 0.06	1.06 ± 0.13	1.11 ± 0.17
CAT	1.00 ± 0.13	0.94 ± 0.12	0.86 ± 0.13
GPX1	1.00 ± 0.09a	0.75 ± 0.08b	0.84 ± 0.07b
GPX2	1.00 ± 0.09a	0.85 ± 0.09b	0.93 ± 0.11ab
SOD1	1.00 ± 0.12a	0.72 ± 0.08b	0.94 ± 0.16a
SOD2	1.00± 0.11	1.27 ± 0.16	0.95 ± 0.08

Data are presented as mean ± SEM. The values having different superscript letters were significantly different (*P* < 0.05).

### Nrf2/Keap1 signal

**Figure 3:**
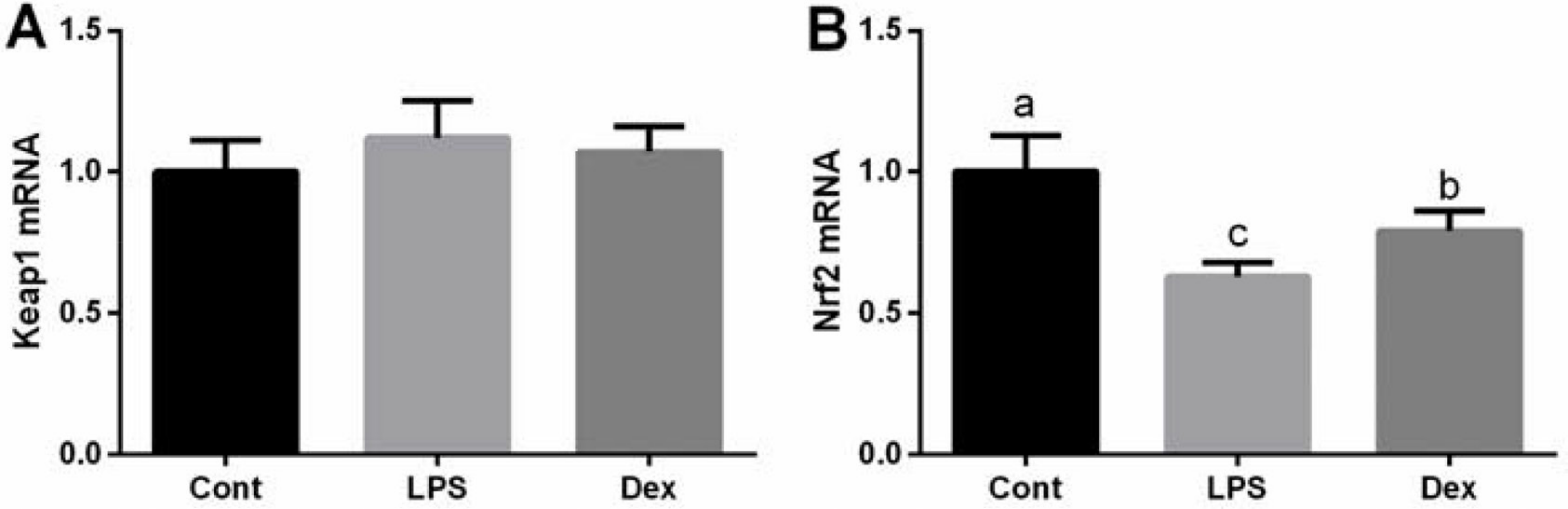
Dexmedetomidine alleviated LPS-induced inhibition of Nrf2 expression in the lung of rats via RT-PCR Data are presented as mean ± SEM. The values having different superscript letters were significantly different (*P* < 0.05).

Nrf2/Keap1 signal has been widely demonstrated to involve in the transcription and expression of antioxidant genes in response to oxidative stress. In this study, we firstly tested Nrf2 and Keap1 expressions in the lung via RT-PCR and found that Nrf2 was markedly downregulated in LPS-challenged rats and dexmedetomidine alleviated Nrf2 inhibition (*P <* 0.05, Figure 3). However, mRNA of Keap1failed to exhibit any significant differences between groups.

**Figure 4:**
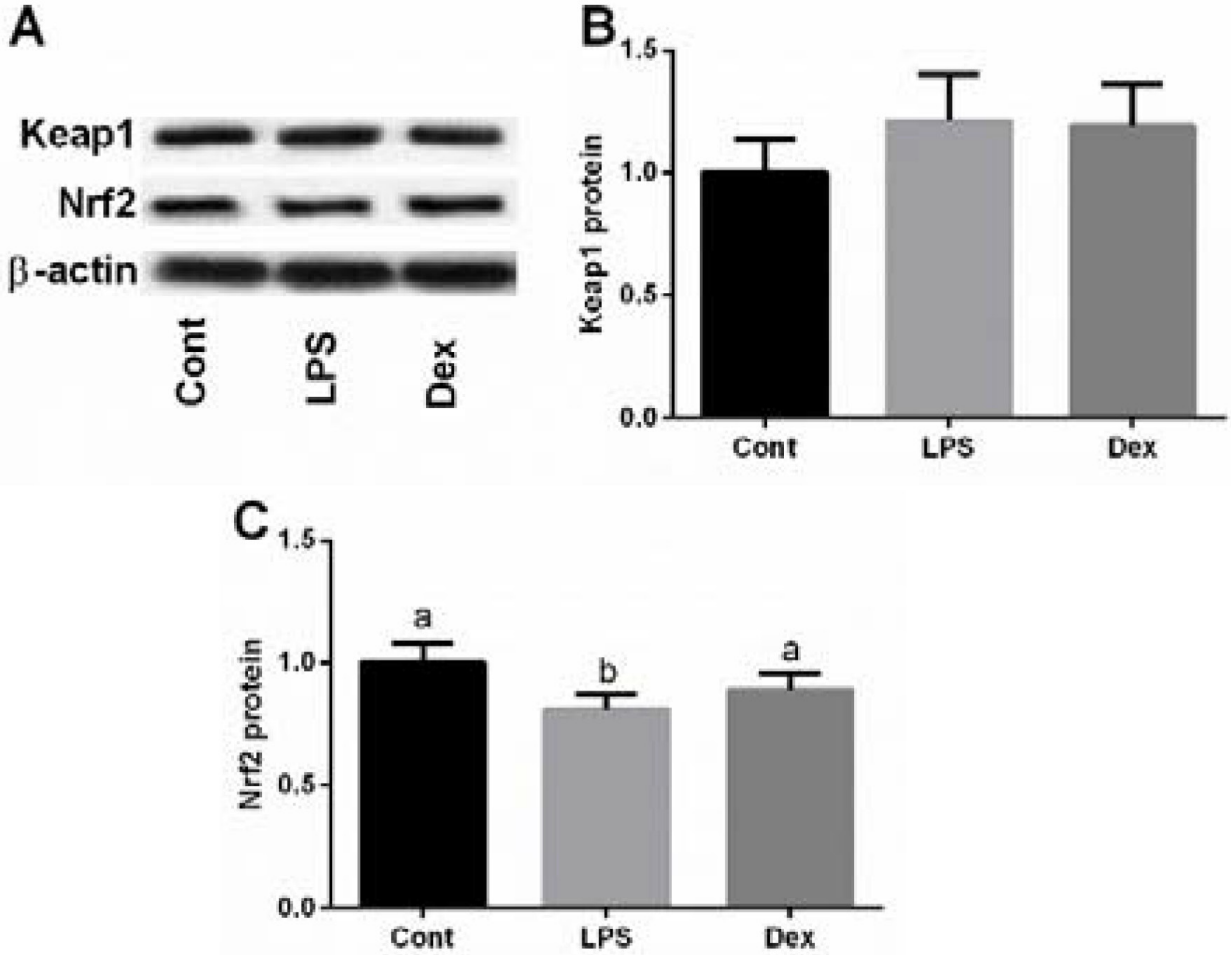
Dexmedetomidine alleviated LPS-induced inhibition of Nrf2 expression in the lung of rats via western blot Data are presented as mean ± SEM. The values having different superscript letters were significantly different (*P* < 0.05).

Protein abundances of Keap1 and Nrf2 were further determined to confirm the effect of dexmedetomidine on LPS-induced inactivation of Nrf2/ Keap1 signal via western blotting analysis (Figure 4A–4C). The results showed that LPS inhibited Nrf2 expression, while dexmedetomidine treatment markedly alleviated LPS-caused Nrf2 inactivation.

### Akt signal

**Figure 5:**
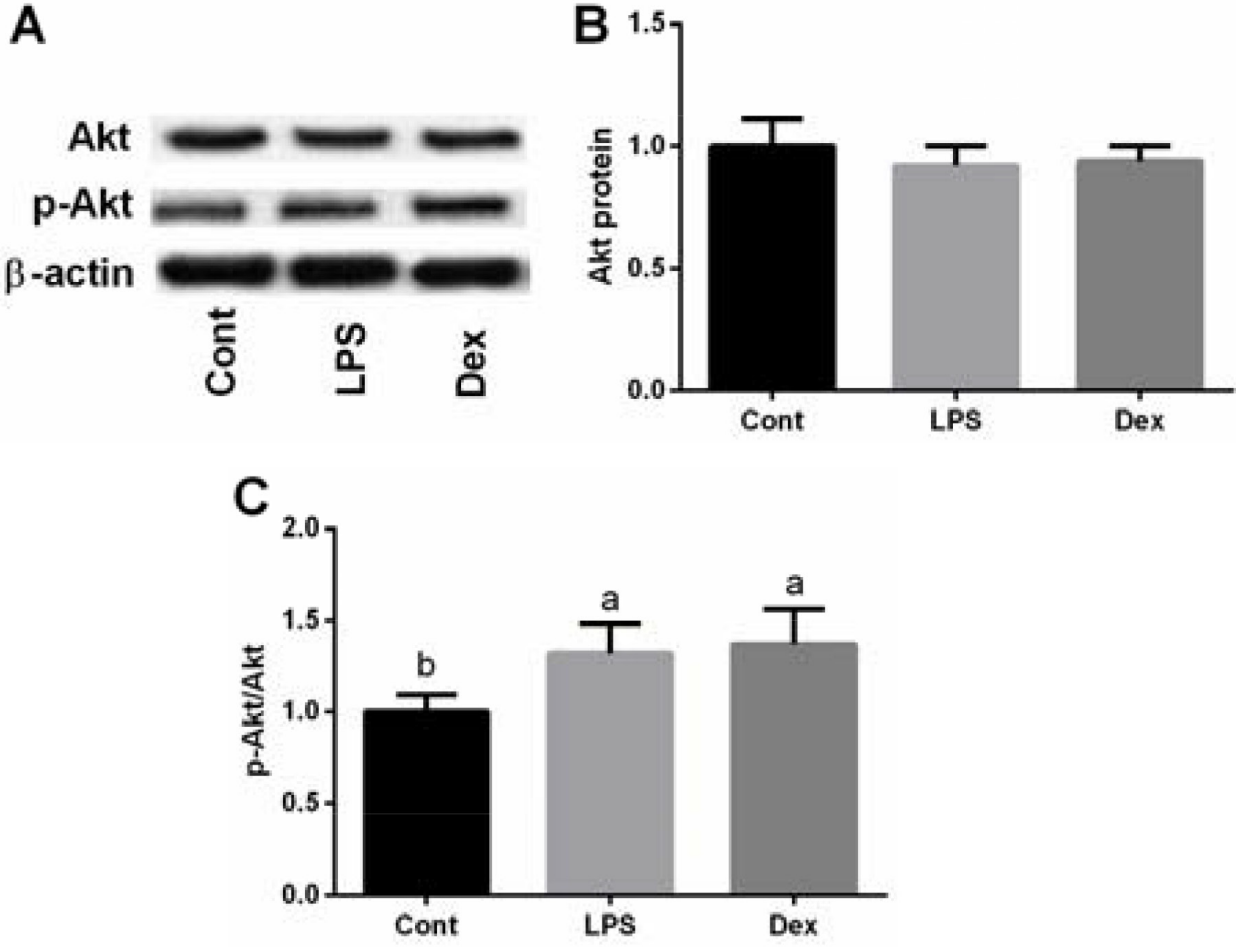
LPS exposure activated Akt siganl in the lung of rats via western blot Data are presented as mean ± SEM. The values having different superscript letters were significantly different (*P* < 0.05).

Total and phosphorylation of Akt in the lung were also tested in this study. Akt phosphorylation ratio was significantly increased in LPS group compared with the control group (*P <* 0.05, Figure 5A–5C), while dexmedetomidine failed to alleviate the inhibitory effect of LPS on Akt phosphorylation ratio (*P* > 0.05).

## DISCUSSION

Inflammatory response associates with neutrophil infiltration, production of pro-inflammatory cytokines, and inflammasomes maturation and plays a critical role in the progression of acute lung injury [9, 10]. In this study, LPS injection induce inflammation in the lung evidenced by the over-expressions of IL-1β, IL-6, IL-17, and TNF-α, while dexmedetomidine treatment alleviated IL-1β and TNF-α expressions. Similarly, Tasdogan et al. reported that a loading dose of dexmedetomidine of one microg/kg over 10 minutes followed by a maintenance dose of 0.2–2.5 microg/kg/h decreased serum TNF-a, IL-1β, and IL-6 levels in patients with severe sepsis after abdominal surgery [11]. Meanwhile, dexmedetomidine as an adjuvant in anesthesia reduced circulating IL-1β, IL-6, TNF-α, and INF-γ levels [12]. These results concluded that dexmedetomidine alleviated LPS-induced inflammation in the lung via mediating IL-1β and TNF-α expressions.

In this study, marked oxidative stress was noticed in rats after exposure evidenced by the increased oxidative products (MDA, 8-OHDG, and HFR) in the serum and lung. Dexmedetomidine treatment reduced serum MDA and lung MDA and HFR generation, suggesting an antioxidant potential in LPS-induced acute lung injury in rats. Dexmedetomidine has been reported to attenuate oxidative stress induced lung alveolar epithelial cell apoptosis via inhibiting reactive oxygen species (ROS) generation [13, 14]. In lung cancer patients during one-lung ventilation, dexmedetomidine pretreatment reduced MDA abundance and enhanced activities of antioxidant enzymes, such as superoxide dismutase (SOD) [15]. In the present study, we failed to monitor the activities of antioxidant enzymes, but expressions of antioxidant genes in the lung were determined and we found that dexmedetomidine treatment significantly upregulated SOD1 expression compared with the LPS group. The activity and expression of SOD1 is essential to maintain cellular ROS under this critical threshold and protect cell against ROS-induced oxidative injury [16–18]. Thus, we speculated that dexmedetomidine upregulated expressions of antioxidant genes, which further protect lung against LPS-induced oxidative stress in rats.

Nuclear factor erythroid 2-related factor 2 (Nrf2)/ Kelch-like ECH-associated inhibitor 1 (Keap1) is a major cytoprotective signal and controls a variety of antioxidant, detoxification, and metabolic genes [19–21]. After activation, conformational changes in the Nrf2/Keap1 complex inhibit proteasomal degradation of Nrf2 and facilitate an increase in Nrf2 translocation into nuclear to bind with antioxidant response element sequences in the promoter regions [17, 19, 22]. Although we failed to found any significant difference in Keap1 expression, Nrf2 was markedly inhibited in response to LPS exposure and dexmedetomidine activated Nrf2 signal compared with LPS-challenged rats. Various studies have confirmed that dexmedetomidine can activate Nrf2 signal to mediate antioxidant genes expressions. For example, dexmedetomidine pretreatment attenuates kidney injury via activating Nrf2 and upregulating antioxidants in rats during orthotopic autologous liver transplantation [23]. In experimental mild acute lung injury, dexmedetomidine improves pulmonary oxygenation and increases Nrf2 expression in the lung tissue [24].

Akt (protein kinase B) is a serine/threonine protein kinase which involves in bacterial infections, LPS tolerance, and expressions of cytokines [25, 26]. Previous reports have demonstrated that Akt signal is activated in LPS-induced acute lung injury and involves in the pathogenesis of acute respiratory failure [27–29], thus targeting Akt signal has been considered as a potential protective strategy for the acute lung injury. However, the present results showed that dexmedetomidine failed to affect Akt signal. In conclusion, Akt signal involves in LPS-induced acute lung injury in rats but Akt fails to involve in the protective mechanism of dexmedetomidine.

## MATERIALS AND METHODS

### Animal model and groups

This study was approved by the animal welfare committee of Shenzhen Bao’an Maternity and Child Health Hospital. Female Wistar rats (10–11 weeks) were randomly assigned into 3 groups (*n* = 8): control group (Cont), LPS-challenged group (LPS), and a LPS plus dexmedetomidine group (Dex). LPS was used to induce acute lung injury in rats via intraperitoneal injection of 10 mg/kg LPS body weight (Sigma, St. Louis, MO, USA). Rats in Dex group received dexmedetomidine intravenously for 90 min at a rate of 5 μg/kg/h/5mL (total dose 7.5 μg/kg) 30min prior to LPS injection. Animals were sacrificed one day after the LPS injection.

### Blood collection and separation

After 24 h, blood samples of all animals were collected from inferior vena cava and centrifuged at 3,500 × g for 15 min to separate serum. Serum samples were stored at –80°C for further analysis.

### Lung collection and preparation

Animals were sacrificed and lung samples were harvested. The left lungs were homogenized (1 g tissue in 9 mL saline) and then centrifuged at 3,000 × g for 10 min under 4 °C. The supernatants were stored at–80 °C for further analysis.

### Oxidative stress

MDA, 8-OHDG, and HFR in the serum and lung supernatants were measured using spectrophotometric kits (Nanjing Jiangcheng Biotechnology Institute, China).

### Real-time PCR

One piece of right lung were harvested and stored at -80 °C. Total RNA of these tissues was isolated with TRIZOL regent (Invitrogen, USA) and reverse transcribed into the first strand (cDNA) using DNase I, oligo (dT) 20 and Superscript II reverse transcriptase (Invitrogen, USA). The reverse transcription was conducted at 37°C for 15 min, 95°C 5 sec. Primers were designed with Primer 5.0 according to the gene sequence of rattus norvegicus to produce an amplification product (Table 3). β-actin was chosen as the house-keeping gene to normalize target gene levels. The PCR cycling condition was 36 cycles at 94°C for 40 sec, 60 °C for 30 sec and 72°C for 35 sec. The relative expression was expressed as a ratio of the target gene to the control gene using the formula 2^-(ΔΔCt)^, where ΔΔCt=(Ct_Target_-Ct_β-actin_)_treatment_-(Ct_Target_-Ct_β-actin_)_control_. Relative expression was normalized and expressed as a ratio to the expression in the control group.

**Table 3:** Primers used in this study

Genes	No.	Nucleotide sequence of primers (5′–3′)	Size (bp)
β-Actin	NM_031144.3	F: CTGTGTGGATTGGTGGCTCT R: CAGCTCAGTAACAGTCCGCC	135
IL-1β	NM_031512.2	F: GACTTCACCATGGAACCCGT R: CAGGGAGGGAAACACACGTT	200
IL-4	NM_201270.1	F: CGTGATGTACCTCCGTGCTT R: ATTCACGGTGCAGCTTCTCA	108
IL-6	NM_012589.2	F: AGCGATGATGCACTGTCAGAA R: GCATTGGAAGTTGGGGTAGGA	281
IL-10	NM_012854.2	F: CCTGGTAGAAGTGATGCCCC R: GATGCCGGGTGGTTCAATTT	281
*IL-17*	NM_001106897.1	F: TCCTCTATTGTCCGCCATGC R: ATTTGTATCCCCTCTGCGCC	194
*TNF-α*	XM_008772775.2	F: AAGCTGTCTTCAGGCCAACA R: CCCGTAGGGCGATTACAGTC	*233*
NQO1	NM_017000.3	F: GGAGACTGTCTGGGAGGAGT R: GCTTTGATCTGGTTGTCGGC	*180*
NQO2	NM_001004214.1	F: TCCAGGAGGCAGAGACTGTT R: AGAGGGCACCAGTAACATCG	*276*
CAT	NM_012520.2	F: TTTTCACCGACGAGATGGCA R: CCCACAAGGTCCCAGTTACC	*274*
GPX1	NM_030826.4	F: AGTGCGAGGTGAATGGTGAG R: GGAATGCCTTAGGGGTTGCT	*280*
GPX2	NM_183403.2	F: GCATGGCTTACATCGCCAAG R: GGAAGCCGAGAACCACTAGG	*201*
SOD1	NM_017050.1	F: AGGGCGTCATTCACTTCGAG R: CCTCTCTTCATCCGCTGGAC	194
SOD2	NM_017051.2	F: ACGCGACCTACGTGAACAAT R: GCCTCCAGCAACTCTCCTTT	196

F: forward; R: reverse; IL: interleukin; TNF-α: tumor necrosis factor; NQO: NAD(P)H quinone dehydrogenase; CAT: catalase; GPX: glutathione peroxidase; SOD: superoxide dismutase

### Western blot

Proteins of lung were extracted with using protein extraction reagents (Thermo Fisher Scientific Inc., USA) and the concentration was tested using BCA protein assay (Sigma-Aldrich, USA). Proteins (30 μg) were separated by SDS–polyacrylamide gel electrophoresis and electrophoretically transferred to a polyvinylidene difluoride (PVDF) membrane (BioRad, Hercules, CA, USA). Membranes were blocked and then incubated with the following primary antibodies: Anti-Keap1 antibody [1F10B6] (ab150654), Anti-Nrf2 antibody (ab31163), Anti-pan-AKT antibody (ab8805), Anti-pan-AKT (phospho T308) antibody (ab38449), anti-Catalase antibody (ab16731), and anti-beta Actin antibody (ab8227). After primary antibody incubation, membranes were washed, incubated with alkaline phosphatase-conjugated anti-mouse or anti-rabbit IgG antibodies (Promega, Madison, WI, USA), and quantified and digitally analyzed using the image J program (NIH).

### Statistical analysis

All data were analyzed by SPSS 17.0 software. Difference was tested by Ducan's multiple comparison test. Data are expressed as the mean ± SEN. Values in the same row with different superscripts are significant (P < 0.05).

## References

[1] Impellizzeri D, Bruschetta G, Esposito E, Cuzzocrea S (2015). Emerging drugs for acute lung injury. Expert opinion on emerging drugs.

[2] Butt Y, Kurdowska A, Allen TC (2016). Acute Lung Injury: A Clinical and Molecular Review. Archives of pathology & laboratory medicine.

[3] Mokra D, Kosutova P (2015). Biomarkers in acute lung injury. Respir Physiol Neurobiol.

[4] Liu CH, Zhang WD, Wang JJ, Feng SD (2016). Senegenin Ameliorate Acute Lung Injury Through Reduction of Oxidative Stress and Inhibition of Inflammation in Cecal Ligation and Puncture-Induced Sepsis Rats. Inflammation.

[5] Wang MT, Liu T, Wang DA, Zheng YH, Wang XD, He J (2011). Therapeutic effects of pyrrolidine dithiocarbamate on acute lung injury in rabbits. J Transl Med.

[6] Cai Y, Xu H, Yan J, Zhang L, Lu Y (2014). Molecular targets and mechanism of action of dexmedetomidine in treatment of ischemia/reperfusion injury (Review). Molecular Medicine Reports.

[7] Geloen A, Pichot C, Leroy S, Julien C, Ghignone M, May CN, Quintin L (2015). Pressor Response to Noradrenaline in the Setting of Septic Shock: Anything New under the Sun-Dexmedetomidine, Clonidine? A Minireview. Biomed Research International.

[8] Zeng XZ, Wang HL, Xing XC, Wang Q, Li WZ (2016). Dexmedetomidine Protects against Transient Global Cerebral Ischemia/Reperfusion Induced Oxidative Stress and Inflammation in Diabetic Rats. Plos One.

[9] Jiang W, Luo F, Lu Q, Liu J, Li P, Wang X, Fu Y, Hao K, Yan T, Ding X (2016). The protective effect of Trillin LPS-induced acute lung injury by the regulations of inflammation and oxidative state. Chem Biol Interact.

[10] Arango D, Diosa-Toro M, Rojas-Hernandez LS, Cooperstone JL, Schwartz SJ, Mo X, Jiang J, Schmittgen TD, Doseff AI (2015). Dietary apigenin reduces LPS-induced expression of miR-155 restoring immune balance during inflammation. Mol Nutr Food Res.

[11] Tasdogan M, Memis D, Sut N, Yuksel M (2009). Results of a pilot study on the effects of propofol and dexmedetomidine on inflammatory responses and intraabdominal pressure in severe sepsis. J Clin Anesth.

[12] Bulow NM, Colpo E, Pereira RP, Correa EF, Waczuk EP, Duarte MF, Rocha JB (2016). Dexmedetomidine decreases the inflammatory response to myocardial surgery under mini-cardiopulmonary bypass. Braz J Med Biol Res.

[13] Cui J, Zhao HL, Wang CY, Sun JJ, Lu KZ, Ma DQ (2015). 1 Dexmedetomidine Attenuates Oxidative Stress Induced Lung Alveolar Epithelial Cell Apoptosis In Vitro. Oxidative Medicine and Cellular Longevity.

[14] Rao PVLNS, Kiranmayi VS, Swathi P, Jeyseelan L, Suchitra MM, Bitla AR (2015). Comparison Of Two Analytical Methods Used For The Measurement Of Total Antioxidant Status. Journal of antioxidant activity.

[15] Gao SQ, Wang YL, Zhao J, Su AP (2015). Effects of dexmedetomidine pretreatment on heme oxygenase-1 expression and oxidative stress during one-lung ventilation. Int J Clin Exp Patho.

[16] van Zundert B, Brown RH (2017). Silencing strategies for therapy of SOD1-mediated ALS. Neuroscience Letters.

[17] de Roos B, Duthie GG (2015). Role of dietary pro-oxidants in the maintenance of health and resilience to oxidative stress. Mol Nutr Food Res.

[18] Sureda A, Bibiloni MD, Martorell M, Buil-Cosiales P, Marti A, Pons A, Tur JA, Martinez-Gonzalez MA, Investigators PS (2016). Mediterranean diets supplemented with virgin olive oil and nuts enhance plasmatic antioxidant capabilities and decrease xanthine oxidase activity in people with metabolic syndrome: The PREDIMED study. Mol Nutr Food Res.

[19] Hybertson BM, Gao B (2014). Role of the Nrf2 signaling system in health and disease. Clin Genet.

[20] Qin S, Hou DX (2016). Multiple regulations of Keap1/Nrf2 system by dietary phytochemicals. Mol Nutr Food Res.

[21] Yan C, Sun W, Wang X, Long J, Liu X, Feng Z, Liu J (2016). Punicalagin attenuates palmitate-induced lipotoxicity in HepG2 cells by activating the Keap1-Nrf2 antioxidant defense system. Mol Nutr Food Res.

[22] Tkachev VO, Menshchikova EB, Zenkov NK (2011). Mechanism of the Nrf2/Keap1/ARE signaling system. Biochemistry (Mosc).

[23] Yu XF, Chi XJ, Wu S, Jin Y, Yao H, Wang YH, Xia ZY, Cai J (2016). Dexmedetomidine Pretreatment Attenuates Kidney Injury and Oxidative Stress during Orthotopic Autologous Liver Transplantation in Rats. Oxidative Medicine and Cellular Longevity.

[24] Cavalcanti V, Santos CL, Samary CS, Araújo MN, Heil LB, Morales MM, Silva PL, Pelosi P, Fernandes FC, Villela N, Rocco PR (2014). Effects of short-term propofol and dexmedetomidine on pulmonary morphofunction and biological markers in experimental mild acute lung injury. Resp Physiol Neurobi.

[25] Lee YG, Lee J, Byeon SE, Yoo DS, Kim MH, Lee SY, Cho JY (2011). Functional role of Akt in macrophage-mediated innate immunity. Front Biosci (Landmark Ed).

[26] Pisonero-Vaquero S, Martinez-Ferreras A, Garcia-Mediavilla MV, Martinez-Florez S, Fernandez A, Benet M, Olcoz JL, Jover R, Gonzalez-Gallego J, Sanchez-Campos S (2015). Quercetin ameliorates dysregulation of lipid metabolism genes via the PI3K/AKT pathway in a diet-induced mouse model of nonalcoholic fatty liver disease. Mol Nutr Food Res.

[27] Yu X, Yu S, Chen L, Liu H, Zhang J, Ge H, Zhang Y, Yu B, Kou J (2016). Tetrahydroberberrubine attenuates lipopolysaccharide-induced acute lung injury by down-regulating MAPK, AKT, and NF-kappaB signaling pathways. Biomed Pharmacother.

[28] Ma C, Zhu L, Wang J, He H, Chang X, Gao J, Shumin W, Yan T (2015). Anti-inflammatory effects of water extract of Taraxacum mongolicum hand.-Mazz on lipopolysaccharide-induced inflammation in acute lung injury by suppressing PI3K/Akt/mTOR signaling pathway. J Ethnopharmacol.

[29] Zhao LL, Hu GC, Zhu SS, Li JF, Liu GJ (2014). Propofol pretreatment attenuates lipopolysaccharide-induced acute lung injury in rats by activating the phosphoinositide-3-kinase/Akt pathway. Braz J Med Biol Res.

